# Qishen Granule Protects against Doxorubicin-Induced Cardiotoxicity by Coordinating MDM2-p53-Mediated Mitophagy and Mitochondrial Biogenesis

**DOI:** 10.1155/2022/4344677

**Published:** 2022-09-06

**Authors:** Weili Li, Yawen Zhang, Xiaoping Wang, Jing Cao, Weina Qian, Guanjing Ling, Nannan Tan, Jinchi Jiang, Qianbin Sun, Chun Li, Wei Wang, Yong Wang

**Affiliations:** ^1^School of Traditional Chinese Medicine, Beijing University of Chinese Medicine, Beijing 100029, China; ^2^Affiliated Hospital of Shaanxi University of Chinese Medicine, Shaanxi University of Chinese Medicine, Xianyang 712000, China; ^3^Modern Research Center for Traditional Chinese Medicine, Beijing University of Chinese Medicine, Beijing 100029, China; ^4^Beijing Key Laboratory of TCM Syndrome and Formula, Beijing University of Chinese Medicine, Beijing 100029, China; ^5^Key Laboratory of TCM Syndrome and Formula (Beijing University of Chinese Medicine), Ministry of Education, Beijing 100029, China; ^6^Guangzhou University of Chinese Medicine, Guangzhou 510006, China

## Abstract

Doxorubicin (DOX), the anthracycline chemotherapeutic agent, is widely used for the treatment of various cancers. However, its clinical application is compromised by dose-dependent and fatal cardiotoxicity. This study is aimed at investigating the cardioprotective effects of Qishen granule (QSG) and the specific mechanism by which QSG alleviates DOX-induced cardiotoxicity (DIC) and providing an alternative for the treatment of DIC. We first evaluated the cardioprotective effects of QSG in a DIC mouse model, and the obtained results showed that QSG significantly protected against DOX-induced myocardial structural and functional damage, mitochondrial oxidative damage, and apoptosis. Subsequently, after a comprehensive understanding of the specific roles and recent developments of p53-mediated mitochondrial quality control mechanisms in DIC, we investigated whether QSG acted on MDM2 to regulate the activity of p53 and downstream mitophagy and mitochondrial biogenesis. The in vivo results showed that DOX inhibited mitochondrial biogenesis and blocked mitophagy in the mouse myocardium, while QSG reversed these effects. Mechanistically, we combined nutlin-3, which inhibits the binding of p53 and MDM2, with DOX and QSG and evaluated their influence on mitophagy and mitochondrial biogenesis in H9C2 cardiomyocytes. The obtained results showed that both DOX and nutlin-3 substantially inhibited mitophagy and mitochondrial biogenesis and induced mitochondrial oxidative damage and apoptosis, which could be partially recovered by QSG. Importantly, the immunoprecipitation results showed that QSG promoted the binding of MDM2 to p53, thus decreasing the level of p53 protein and the binding of p53 to Parkin. Collectively, QSG could promote the degradation of p53 by enhancing the binding of MDM2 to the p53 protein, resulting in the reduced binding of p53 to the Parkin protein, thus improving Parkin-mediated mitophagy. Increased degradation of p53 protein by QSG simultaneously enhanced mitochondrial biogenesis mediated by PGC-1*α*. Ultimately, QSG relieved DOX-induced mitochondrial oxidative damage and apoptosis by coordinating mitophagy and mitochondrial biogenesis.

## 1. Introduction

Doxorubicin, classified as an anthracycline, is widely used in the clinic to treat numerous solid tumours, lymphomas, and leukaemias, due to its high efficiency and wide spectrum [1, 2]. However, despite its potency, the clinical benefit of DOX is largely limited by the risk of dose-dependent cardiotoxicity, which is characterized by a reduction in the left ventricular ejection fraction and eventually congestive heart failure [3]. Dexrazoxane is the only FDA-approved cardioprotectant for patients with anthracycline chemotherapy [4]. Given multiple undesirable side effects, such as bone marrow suppression, hepatotoxicity, and secondary malignancy occurrence [5], there is a pressing need for advancements in safe and effective adjuvant drug discovery to attenuate DOX-induced cardiotoxicity.

Understanding the molecular basis of DIC will provide novel insights for the treatment of DIC. The prevailing view about the mechanistic underpinnings of DIC is that DOX induces excessive reactive oxygen species (ROS) production, resulting in mitochondrial DNA damage, lipid peroxidation in membrane structures such as the mitochondrial membrane, defects in the respiratory chain/oxidative phosphorylation (OXPHOS) system, and eventually mitochondrial dysfunction [6–8]. Accordingly, the maintenance of a healthy and functional mitochondrial population is critical to cope with physiological and pathological stress. Mitochondria have developed finely tuned mechanisms of quality control, in which mitophagy targets mitochondria for the removal of damaged mitochondria or their components, while mitochondrial biogenesis consists of the addition of new proteins and/or lipids to the preexisting mitochondrial reticulum, orchestrating the biogenesis, maintenance, and turnover of mitochondria [9–11]. Therefore, the modulation of key parameters controlling the balance between mitophagy and mitochondrial biogenesis is a promising approach to maintain functional mitochondria.

p53 is a critical protein for inducing apoptosis or cell cycle arrest in response to DNA damage and cellular stress [12–14]. Accumulating evidence has demonstrated that p53 is activated in DOX-treated hearts and in vitro cardiomyocytes [15–17]. Among them, Sahin et al. verified that MEFs or heart tissues of mice treated with DOX showed increased p53 levels and coincident binding of p53 to the PGC-1*α* promoter and repression of PGC-1*α* levels. PGC-1*α* plays a central role in a regulatory network positively governing the transcriptional control of mitochondrial biogenesis and respiratory function [18]. Additionally, Hoshino et al. documented that activated cytosolic p53 binds to Parkin and disturbs its translocation to damaged mitochondria and their subsequent clearance by mitophagy in DOX-treated hearts [19]. Together, p53 may be the common culprit of DOX-induced mitochondrial biogenesis and mitophagy disorders, and regulating the function and activity of p53 has become an effective strategy to treat DOX-induced mitochondrial dysfunction. MDM2 was initially discovered as an inhibitor of p53 through binding with p53 and blocking the binding of p53 to its targeted DNA or ubiquitinating p53 for proteasomal degradation [20–23]. The pharmacologic amplification of the p53-MDM2 interaction represents an attractive approach for the treatment of DIC.

The traditional Chinese medicine (TCM) formula Qishen granule (QSG) consists of six herbs: Radix Astragali mongolici, Radix Salvia miltiorrhizabunge, Flos Lonicerae, Radix Scrophulariae, Radix Aconiti Lateralis Preparata, and Radix Glycyrrhizae. It was developed from the traditional formula “Zhen-Wu-Tang (ZWT),” which is widely prescribed by TCM doctors for the treatment of heart disease and has been repeatedly reported to improve heart function and reduce fibrosis [24–26]. However, its cardioprotective role and mechanism in DIC have not been determined thus far. Therefore, this study is aimed at investigating whether QSG can alleviate DOX-induced cardiac dysfunction and mitochondrial dyshomeostasis and maintain mitochondrial quality and function through mitophagy and mitochondrial biogenesis regulated by the MDM2-p53 pathway.

## 2. Materials and Methods

### 2.1. Animals and Pharmacological Treatments

C57BL/6 wild-type mice (20 g ± 2 g) were obtained from Beijing SPF Biotechnology (Beijing, China). All animal experimental procedures and care were authorized by Beijing University of Chinese Medicine Animal Care Committee and performed following the Guide for the Institutional Animal Care and Use Committee. The animals were housed in a 12 : 12 h light-dark cycle temperature-controlled environment (22°C) and allowed free access to standard laboratory chow and water. A DIC mouse model in this study was generated as described previously [27–29]. The mice were randomly separated into 6 groups of 9 mice each based on the following treatment regimens: mice in the DOX group, high, medium, and low doses of QSG (H-QSG, M-QSG, and L-QSG) group, and enalapril (ENA) group were injected into the tail vein with DOX (5 mg/kg) once weekly for 4 consecutive weeks, and mice in the control group were treated with equal volumes of saline solution (0.9% NaCl). One week after the last injection of DOX or saline solution, QSG (6.66 g/kg, 3.33 g/kg, and 1.67 g/kg) and ENA (15 mg/kg) dissolved in ultrapure water were given by gavage once a day for 4 weeks. The mice in the control and DOX groups were given the same volume of ultrapure water. 3.33 g/kg of QSG administered to mice was clinical equivalent dose, as described before [30]. Further, we set the one-half and two-fold of 3.33 g/kg QSG to low and high doses, respectively. Enalapril has been reported to have dramatic cardioprotective effects against DIC [31], and its dose is roughly distributed in 10-20 mg/kg; thus, we used 15 mg/kg ENA as a positive control in mouse experiments.

### 2.2. Cell Culture and Pharmacological Treatments

H9C2 cells were cultured in DMEM supplemented with 10% FBS at 37°C in a humidified environment containing 5% CO_2_. We referred to 1 *μ*M DOX in a previous study [27–29] as the working concentration used in this study. H9C2 cells were pretreated with a corresponding concentration of QSG for 24 h before cotreatment with 1 *μ*M DOX for 24 h with or without QSG (1~1000 *μ*g/mL). Subsequently, cell viability was determined. Moreover, nutlin-3, an inhibitor of the MDM2-p53 interaction, was used as a positive control of DOX and given to H9C2 cells at a dose of 20 *μ*M.

### 2.3. Assessment of Cardiac Structure and Functions by Echocardiography, Serum CK-MB, LDH, and MDA Detection, and Histological Examination

After 28 days of drug treatment of mice, echocardiography was performed in M-mode with a VEVO 2100 echocardiography system (Visual Sonics, Toronto, ON, Canada) as described previously [27]. Left ventricular end-diastolic dimension (LVEDD), left ventricular end-systolic dimension (LVESD), left ventricular end-systolic volume (LVESV), and left ventricular end-diastolic volume (LVEDV) were measured using computer algorithms. Then, the mice were sacrificed, and the heart and blood were collected. The sera were isolated to measure creatine kinase isoenzymes (CK-MB), lactate dehydrogenase (LDH), and malonaldehyde (MDA). CK-MB was analyzed and determined by automatic biochemical analyzer. LDH and MDA were measured in accordance with the kit instructions (Nanjing Jiancheng Bioengineering Institute, Jiangsu, China), respectively, and the absorbance of the standard substance and the sample to be tested was determined. Finally, the contents of LDH and MDA in each serum sample were calculated. The hearts of mice were immobilized with 4% paraformaldehyde, embedded in paraffin, sectioned into 4 *μ*m serial sections, stained with haematoxylin-eosin (H&E), and observed for morphological changes under an optical microscope at 200x magnification.

### 2.4. Transmission Electron Microscopy (TEM)

Cardiac tissue samples were harvested from mice, immediately rinsed in PBS, and then immersed in 2.5% glutaraldehyde for 2 h at room temperature. The following process was performed as described previously [29]. Images were acquired under an electron microscope (Leica, Buffalo Grove, IL, USA). Mitochondrial autophagosomes were randomly counted based on 6 images from various fields of view (2,500x magnification).

### 2.5. Cell Death Analysis

The apoptotic levels of heart tissue cells were determined by terminal deoxynucleotidyl transferase-mediated nick end labelling (TUNEL) following the manufacturer's instructions. TUNEL was performed on cardiac tissue sections, followed by staining with anti-*α*-actin antibody to label cardiomyocytes and 4,6-diamidino-2-phenylindole (DAPI) to label nuclei. To determine the ratio of living and dead cardiomyocytes in vitro, H9C2 cells were incubated with calcein and propidium iodide (PI) for 30 min at 37°C in the dark. Images were randomly obtained using an optical microscope at 200x and 400x magnification.

### 2.6. ROS Detection

MitoSOX™ Red and dihydroethidium (DHE) staining were applied separately to detect the formation of mitochondrial superoxide in H9C2 cells and fresh heart tissue. Briefly, pretreated H9C2 cells were collected and later incubated with MitoSOX™ Red (5 *μ*M) for 20 min at 37°C in the dark for fluorescence imaging. Fresh frozen sections of cardiac tissue were incubated with DHE (5 *μ*M) for 30 min at 37°C in a light-protected humidified chamber, followed by staining with DAPI to label nuclei. The images were acquired with a laser scanning confocal microscope at 200x magnification.

### 2.7. Mitochondrial Membrane Potential

5,5′,6,6′-Tetrachloro-1,1′,3,3′-tetraethylbenzimidazolyl-carbocyanine iodide (JC-1, Beijing Solaibao Technology Co., Ltd., Beijing, China) was used to monitor the mitochondrial membrane potential (ΔΨ*m*). JC-1 is a cationic dye that can accumulate in the mitochondria in a potential-sensitive manner. JC-1 is a monomer that emits green fluorescence at low ΔΨ*m*. However, at high ΔΨ*m*, the dye aggregates and emits red fluorescence. After pharmacological treatments, the cells were directly collected and later incubated with a 1× JC-1 working solution for 30 min at 37°C in the dark for fluorescence-activated cell sorting on a FACS Canto II flow cytometer (BD Biosciences). ΔΨ*m* was indicated by the ratio of red/green fluorescence intensity.

### 2.8. Adenosine Triphosphate (ATP) Content

ATP levels in myocardial tissue and H9C2 cells were measured using an ATP Assay Kit (Beyotime Biotechnology, Shanghai, China) according to the manufacturer's instructions. Briefly, myocardial tissue or H9C2 cells were lysed in ATP assay buffer and centrifuged at 12,000 g at 4°C for 10 min. Then, 20 *μ*L of the supernatant was added to 100 *μ*L of luciferase reagent in 96-well plates in the dark for the detection of ATP, and the emitted light was measured using a microplate reader. Finally, the concentration was normalized to the protein concentration.

### 2.9. Quantification of mtDNA Copy Number

Total DNA of heart tissues and H9C2 cells was extracted using a DNeasy Blood & Tissue Kit (Qiagen, Hilden, Germany) in accordance with the manufacturer's instructions. The mitochondrial DNA copy number in H9C2 cells was determined by short-range PCR (SRPCR) using FastStart Universal SYBR Green Master Mix (Rox) and then normalized to GAPDH. Primers for GAPDH were purchased from Sangon Biotech (Shanghai) Co., Ltd. SRPCR primers amplified the mtDNA in the 12S rRNA coding region, and the primer sequences were as follows in Table 1.

### 2.10. Immunofluorescence Staining

After drug treatment, H9C2 cells seeded on confocal dishes were stained with MitoTracker Red prior to fixation with 4% paraformaldehyde and subsequently permeabilized with 0.2% Triton X-100. First, the cells were blocked with 5% BSA blocking buffer for 1.5 h and then stained with the primary antibody at 4°C overnight. Finally, the cells were incubated with a second antibody for 1 h at 37°C, and nuclei were counterstained with DAPI for 15 min at 37°C. Similarly, fresh and frozen cardiac tissue sections were permeabilized, blocked, and stained with primary and secondary antibodies and incubated with DAPI as described above. Images were acquired and analysed under a laser scanning confocal microscope.

### 2.11. Mt-mKeima Adenovirus Transfection

The transfection of mt-mkeima adenovirus was performed in accordance with the instructions provided by Hanbio Technology Co., Ltd., Shanghai, China. Briefly, H9C2 cells grown on confocal dishes were transfected with mt-mkeima adenovirus at a multiplicity of infection (MOI) of 50 at 37°C. After incubation for 6 h, the medium was changed to fresh medium containing the drugs. The cells were observed by a confocal microscope. Mt-mkeima, a pH-sensitive fluorescent protein, shifts the excitation spectrum from 440 to 586 nm when mitochondria are delivered to acidic lysosomes, appearing as a shift from green to red. Each cell was evaluated for the number of green and red puncta to monitor the mitophagy flux.

### 2.12. Coimmunoprecipitation (Co-IP) Assay

Co-IP assays were performed according to the manufacturer's instructions for the immunoprecipitation assay kit (Cell Signaling Technology, United States). First, the cell extract was incubated with anti-p53 antibody or control IgG1 antibody at 4°C overnight. Then, Protein G magnetic beads were added to the samples and incubated at 4°C for 30 min. Immunoprecipitates were collected by magnetic separation and washed with buffers 5 times. Finally, the precipitates were treated with SDS buffer solution, and the samples were heated to 98°C for 5 min for subsequent immunoblot analysis.

### 2.13. Immunoblot Analysis

The processes were performed as described previously [29]. The antibodies used in the study are shown in Supplemental Table 1.

### 2.14. Statistical Analysis

Statistical analysis was performed using GraphPad software 6. All results are expressed as the mean ± s.d. The statistical significance of the differences between the means was evaluated using a two-tailed unpaired Student's *t*-test. Alternatively, one-way analysis of variance (ANOVA) was used to assess the differences for more than two groups. A *P* value < 0.05 was considered statistically significant.

## 3. Results

### 3.1. QSG Attenuated DOX-Induced Structural and Functional Lesions in the Heart

**Figure 1:**
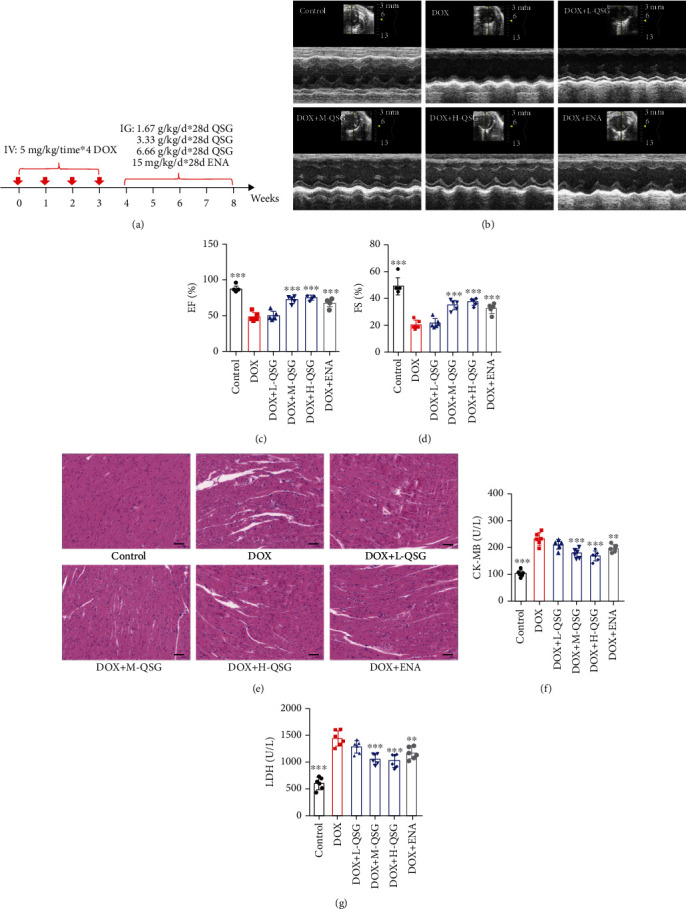
The cardioprotective effects of QSG on DIC mice. (a) A schematic diagram of animal model generation and drug administration. (b–d) Representative images of echocardiograms and quantitative statistics of left ventricular ejection fraction and fractional shortening (*n* = 6). (e) Representative images of morphological and structural changes in the myocardium detected by HE staining. Scale bar: 50 *μ*m. (f, g) The content of serum CK-MB and LDH (*n* = 6). ^∗∗^*P* < 0.01, ^∗∗∗^*P* < 0.001 vs. DOX group. The data are shown as the mean ± s.d.

The protocol for DIC mouse model construction and drug administration is shown in Figure 1(a). After four weeks of administration, cardiac function was evaluated by echocardiography. As shown in Figures 1(b)–1(d), DOX resulted in significantly lower EF and FS values in mice than in the control group. However, EF and FS values treated with medium and high doses of QSG significantly increased compared with the DOX group. Enalapril also increased the EF and FS values of DIC mouse hearts. Consistent with changes in cardiac function, DOX induced disordered arrangement of cardiac tissue, intercellular space expansion, cell loss, and inflammatory cell infiltration, which were alleviated by low, medium, and high doses of QSG and enalapril. In addition, the contents of CK-MB and LDH in serum, critical markers of myocardial injury, increased in the DOX group compared to those in the control group, and QSG dose-dependently reduced their contents (Figures 1(f) and 1(g)).

### 3.2. QSG Attenuated DOX-Induced Mitochondrial Oxidative Damage and Apoptosis in the Mouse Myocardium

**Figure 2:**
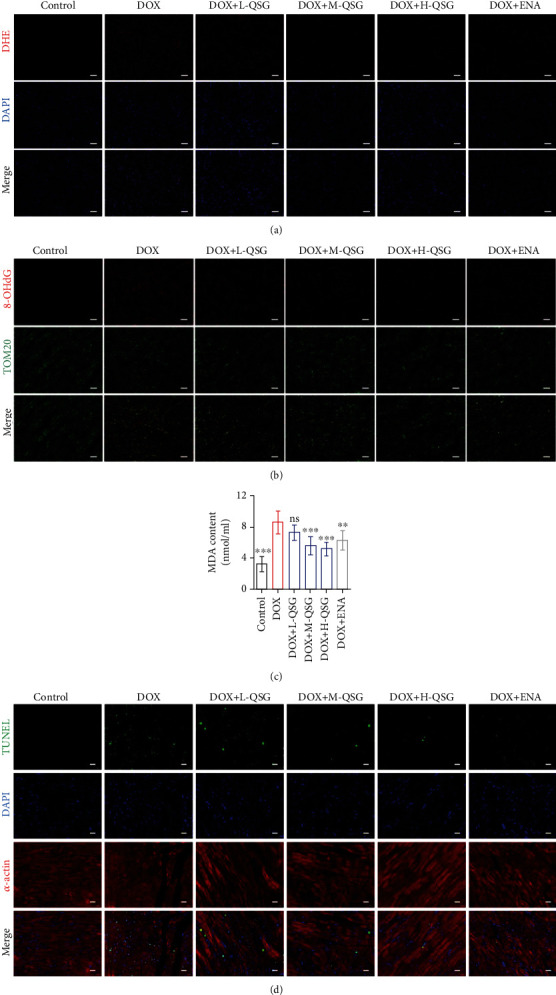
QSG attenuated DOX-induced mitochondrial oxidative damage and apoptosis in the mouse myocardium. (a) Representative images of ROS by DHE staining of mouse myocardium. Scale bar: 50 *μ*m. (b) Representative images of mitochondrial DNA (mtDNA) damage by 8-OHdG and TOM20 immunofluorescence costaining in mouse myocardium. Scale bar: 50 *μ*m. (c) Serum MDA content (*n* = 6). (d) Representative images of apoptosis by TUNEL in mouse myocardium. Scale bar: 20 *μ*m. ^∗∗^*P* < 0.01, ^∗∗∗^*P* < 0.001 vs. DOX group. The data are shown as the mean ± s.d.

Currently, it is well-recognized that ROS-induced mitochondrial damage ultimately leads to cell death as the major cause of DOX-induced cardiotoxicity [6]. Consistently, the results reported in this section also confirmed that DOX could induce severe mitochondrial oxidative damage and apoptosis. First, as shown in Figure 2(a), DOX induced excessive ROS production, as shown by a surge in the number of red fluorescent dots in the mouse myocardium compared to the control group. As the dose of QSG increased, ROS production proportionally decreased. In addition, it has been reported that excessive ROS production could induce subsequent mitochondrial DNA damage and lipid peroxidation of membrane structures, which was consistent with our results, while QSG decreased the production of 8-OHdG represented by a decreased number of red fluorescent dots and MDA at different dosages (Figures 2(b) and 2(c)). Moreover, we found that DOX induced a surge in cardiomyocyte apoptosis represented by an increased number of green fluorescent dots, and the apoptosis rate gradually decreased with increasing QSG dose (Figure 2(d)).

### 3.3. QSG Alleviated DOX-Induced Mitochondrial Biogenesis Depression in the Mouse Myocardium

**Figure 3:**
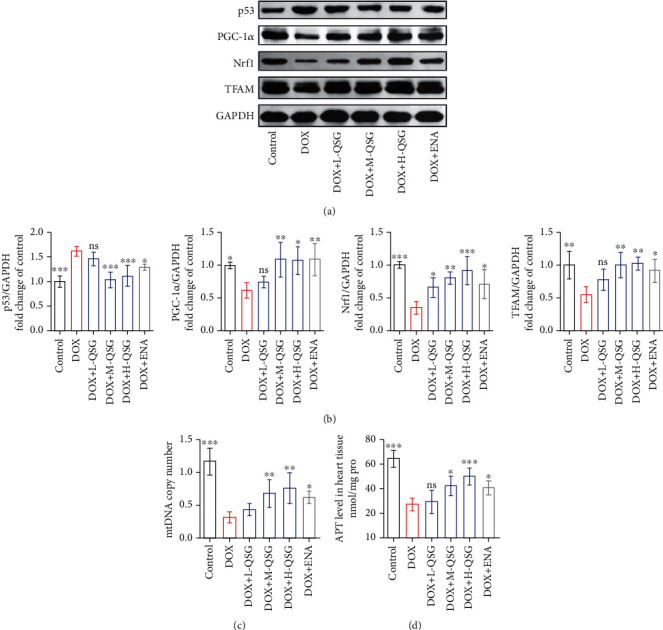
QSG alleviated DOX-induced mitochondrial biogenesis depression in the mouse myocardium. (a, b) Representative immunoblots and quantitative analysis of the expression of p53-mediated mitochondrial biogenesis-related proteins. The expression levels were normalized to GAPDH (*n* = 4). (c) Quantitative detection of mitochondrial DNA (mtDNA) copy number in mouse myocardium by qPCR (*n* = 5). (d) The ATP content in the mouse myocardium (*n* = 5). ^∗^*P* < 0.05, ^∗∗^*P* < 0.01, ^∗∗∗^*P* < 0.001 vs. DOX group. The data are shown as the mean ± s.d.

PGC-1*α*, as a transcriptional coactivator, regulates mitochondrial biogenesis, including mitochondrial mass and function, in response to cellular metabolic stress, such as the constant production of ROS. Moreover, it has been reported that increased p53 induced by DOX could bind to the PGC-1*α* promoter and repress the expression of PGC-1*α*. Thus, we validated the intervention of QSG and DOX on p53-PGC-1*α*-mediated mitochondrial biogenesis in the mouse heart. Impressively, DOX increased the protein level of p53 in the mouse myocardium, while PGC-1*α* and its downstream transcriptionally regulated protein levels of Nrf1 and TFAM in the mouse myocardium were significantly reduced in the DOX group compared with the control group. Low, medium, and high doses of QSG and ENA significantly reduced the protein level of p53 and increased the protein levels of PGC-1*α*, Nrf1, and TFAM (Figures 3(a) and 3(b)). In addition, DOX resulted in a sharp decrease in mitochondrial DNA copy number and ATP production in mouse cardiomyocytes, while QSG increased mitochondrial DNA copy number and ATP production with increasing dose (Figures 3(c) and 3(d)).

### 3.4. QSG Countered DOX-Induced Mitophagy Inhibition in Mouse Myocardium

**Figure 4:**
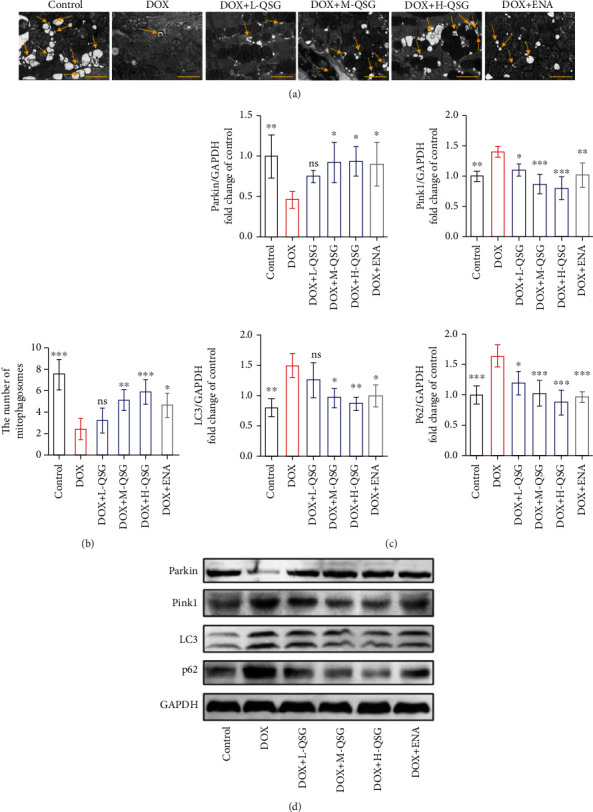
QSG relieved DOX-induced mitophagy inhibition in the mouse myocardium. (a, b) Representative electron micrographs of the heart and the quantification of mitophagosomes (*n* = 6). Scale bar: 2 *μ*m. (c, d) Representative immunoblots and quantitative analysis of the expression of Parkin-mediated mitophagy-related proteins. The expression levels were normalized to GAPDH (*n* = 3-4). ^∗^*P* < 0.05, ^∗∗^*P* < 0.01, ^∗∗∗^*P* < 0.001 vs. DOX group. The data are shown as the mean ± s.d.

Mitophagy, a mitochondrial quality control mechanism, selectively removes damaged mitochondria to maintain a healthy mitochondrial population and relieves mitochondrial damage stress. Therefore, we investigated whether QSG could regulate mitophagy, especially Parkin-mediated mitophagy, to alleviate DOX-induced mitochondrial damage stress in DIC mouse hearts. Substantially reduced mitophagosomes were observed in the DOX group compared with the control group, while progressively increased mitophagosomes were visible in the low, medium, and high doses of QSG groups (Figures 4(a) and 4(b)). Furthermore, we detected changes in Parkin-mediated mitophagy-related protein levels in different groups and found that the levels of Pink1, LC3, and P62 proteins were increased except for decreased Parkin protein in the DOX group compared with those in the control group, which was reversed by low, medium, and high doses of QSG and ENA (Figures 4(c) and 4(d)). In summary, we concluded that DOX may inhibit the expression of Parkin protein in the mouse myocardium, thereby reducing the formation of mitophagosomes, resulting in the inhibition of the degradation of other participating proteins, including Pink1, LC3, and P62, and the elevation of these protein levels.

### 3.5. QSG Regulated Parkin-Mediated Mitophagy by Interfering with the Protein Binding of p53, MDM2, and Parkin in H9C2 Cells

The obtained results of CCK8 suggested that 1-500 *μ*g/mL QSG had little inhibition on H9C2 cell viability, and even 1000 *μ*g/mL QSG markedly improved cell viability. Moreover, 500-1000 *μ*g/mL QSG significantly recovered the inhibition of DOX on cell viability; thus, 500 *μ*g/mL QSG was applied for subsequent experiments (Supplemental Figure 1).

**Figure 5:**
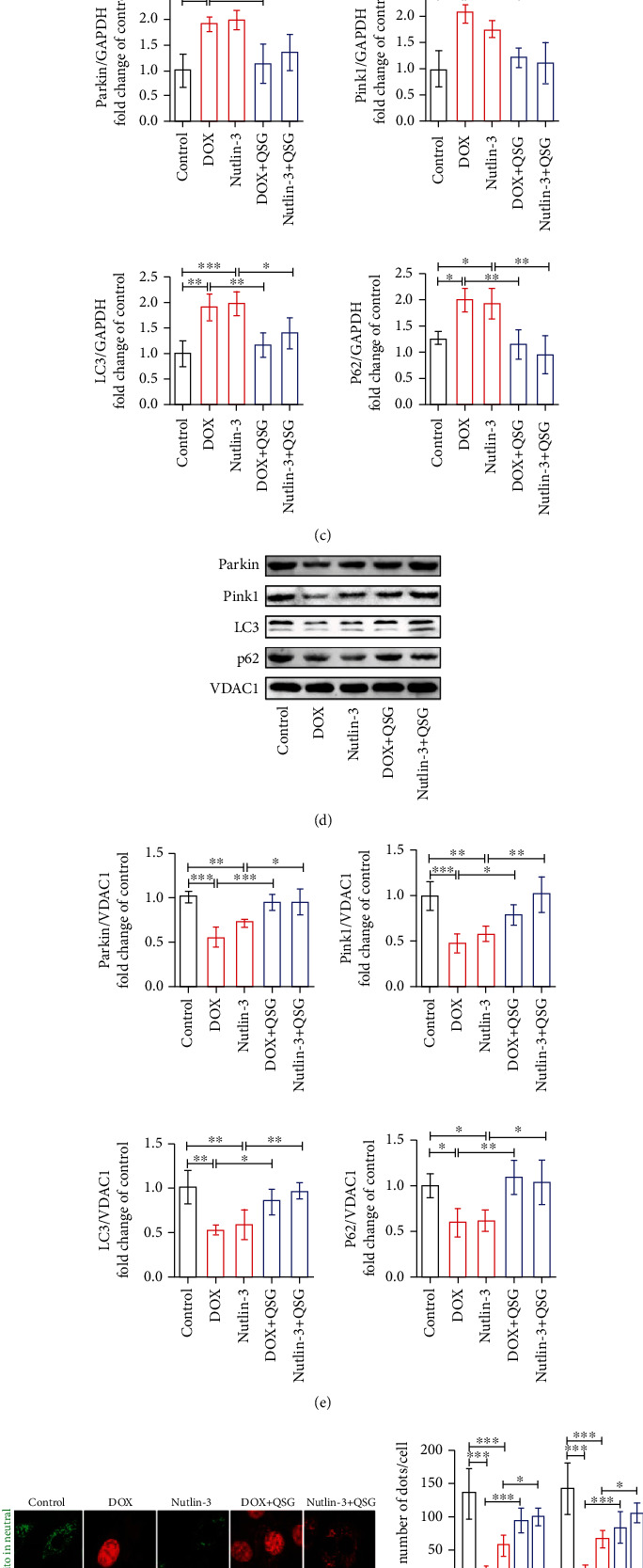
QSG regulated Parkin-mediated mitophagy by interfering with the protein binding of p53, MDM2, and Parkin in H9C2 cells. (a) The detection of the protein binding of p53, MDM2, and Parkin in H9C2 cells by immunoprecipitation. (b, c) Representative immunoblots and quantitative analysis of the expression of Parkin-mediated mitophagy-related proteins in the cytoplasm. The expression levels were normalized to GAPDH (*n* = 4). (d, e) Representative immunoblots and quantitative analysis of the expression of Parkin-mediated mitophagy-related proteins in mitochondria. The expression levels were normalized to VDAC1 (*n* = 4). (f, g) Quantitative analysis of mitophagy flow by transfection of mt-mkeima adenovirus into H9C2 cells. ^∗^*P* < 0.05, ^∗∗^*P* < 0.01, ^∗∗∗^*P* < 0.001 vs. DOX or Nutlin-3 group (*n* = 4). The data are shown as the mean ± s.d.

We have documented that QSG may alleviate DOX-induced mitochondrial oxidative damage and maintain mitochondrial health and function by recalling mitophagy in DIC mouse myocardium. However, the molecular mechanism is still uncertain. Therefore, in this section, we continue to verify the possible mechanism by which QSG regulates mitophagy in H9C2 cardiomyocytes. MDM2 has risen to some prominence because it inhibits p53 function by multiple mechanisms mediated by their direct protein–protein interaction, resulting in reduced binding of p53 to DNA and increased degradation of p53 [23]. In addition, it has been reported that p53 binds to Parkin in the cytoplasm, thereby inhibiting Parkin's transfer to mitochondria, leading to mitophagy blockage [19, 32]. In view of the abovementioned reports on p53, we are interested in the molecular mechanism by which DOX and QSG regulate the interactions of MDM2, Parkin, and p53 proteins in H9C2 cardiomyocytes and whether they ultimately affect mitophagy. Thereafter, p53 protein was upregulated in the DOX group, and increased protein–protein binding of p53 to MDM2 or Parkin was also observed in the DOX group. In QSG-treated H9C2 cells, decreased p53 and MDM2 protein levels and protein binding of Parkin to p53 were detected, while the binding of MDM2 to p53 was significantly increased (Figure 5(a)). These results suggested that QSG promoted the binding of p53 and MDM2 proteins and the degradation of p53 protein, resulting in the reduced binding of p53 to Parkin protein. Subsequently, we applied nutlin-3, a p53 agonist that inhibits the binding of p53 to the MDM2 protein, to determine whether the binding of MDM2 and p53 proteins affects Parkin-mediated mitophagy. We found that DOX and nutlin-3 significantly reduced the levels of mitophagy proteins, including Parkin, Pink1, LC3, and P62, in mitochondria and increased the distribution of these proteins in the cytoplasm compared to the control group (Figures 5(b)–5(e)). QSG partially reversed the above effects of both DOX and nutlin-3 on mitophagy proteins (Figures 5(b)–5(e)). In Figure 5(f), the green puncta of mt-mkeima represent the sum of mitophagosomes and mitochondria not involved in mitophagy, while red puncta represent the mitophagosomes incorporated by the autolysosome. DOX and nutlin-3 reduced the number of all mitophagosomes and mitochondria not involved in mitophagy and mitophagolysosomes, while QSG partially reversed these effects again. In conclusion, these results suggested that DOX induced a p53 expression surge and increased p53 binding to Parkin protein in H9C2 cardiomyocytes, resulting in reduced Parkin transfer to mitochondria and inhibition of Parkin-mediated mitophagy; nutlin-3 inhibited the distribution of Parkin and other mitophagy proteins in mitochondria and the occurrence of mitophagy, which may be caused by the fact that nutlin-3 enhanced the binding of free p53 and Parkin protein while inhibiting the binding of p53 and MDM2 protein; QSG reduced the binding of free p53 to Parkin protein by promoting the binding of p53 to MDM2 protein for the degradation of p53, thus increasing the transfer of Parkin to mitochondria and subsequent occurrence of Parkin-mediated mitophagy.

### 3.6. QSG Enhanced Mitochondrial Biogenesis by Inhibiting p53 in H9C2 Cells

**Figure 6:**
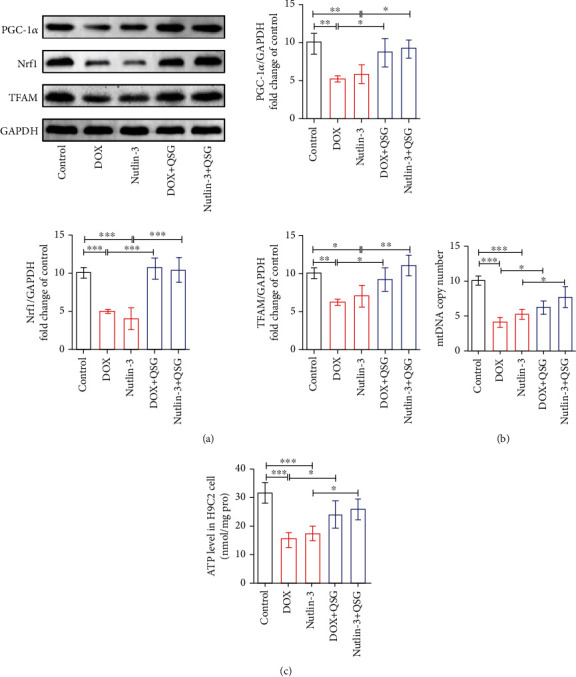
QSG enhanced mitochondrial biogenesis by inhibiting p53 in H9C2 cells. (a) Representative immunoblots and quantitative analysis of the expression of p53-mediated mitochondrial biogenesis-related proteins. The expression levels were normalized to GAPDH (*n* = 4). (b) Quantitative analysis of mtDNA copy number in H9C2 cells by qPCR (*n* = 4). (c) Quantitative analysis of ATP content in H9C2 cells (*n* = 4). ^∗^*P* < 0.05, ^∗∗^*P* < 0.01, ^∗∗∗^*P* < 0.001 vs. DOX or Nutlin-3 group. The data are shown as the mean ± s.d.

To elucidate whether QSG enhanced mitochondrial biogenesis through the p53-PGC-1*α* signalling axis in DIC mouse hearts, we applied nutlin-3, which activated the function of p53 by inhibiting the binding of p53 to MDM2, to H9C2 cells to further evaluate the changes in mitochondrial biogenesis proteins, mitochondrial mass, and function. As illustrated in Figure 6(a), after nutlin-3 treatment, the expression levels of PGC-1*α* and its downstream proteins Nrf1 and TFAM significantly decreased in H9C2 cardiomyocytes, and DOX also inhibited these proteins. These results suggested that nutlin-3 inhibited the binding of p53 to MDM2 and restored the activity of p53, and DOX activated p53 protein expression, both of which inhibited the expression of PGC-1*α* and other mitochondrial biogenesis proteins. However, QSG rescued the perturbations of DOX and nutlin-3 centring on PGC-1*α* and its downstream proteins Nrf1 and TFAM by enhancing the protein binding between p53 and MDM2 and then restraining the expression of p53 protein (Figures 5(a) and 6(a)). Consistently, mitochondrial DNA replication and ATP production coordinated by PGC-1*α* were reduced in the DOX and nutlin-3 groups, and QSG partially restored these mitochondrial mass and function deficits (Figures 6(b) and 6(c)). The above profiles suggested that QSG restrained the function of p53 by both inhibiting the expression of p53 protein and enhancing the binding of p53 to MDM2 protein, resulting in improvement in the mitochondrial biogenesis mediated by PGC-1*α*, which is manifested as a marked increase in mitochondrial biogenesis-related proteins, mitochondrial copy number, and ATP.

### 3.7. QSG Reduced DOX-Induced Mitochondrial Oxidative Damage and Apoptosis by Suppressing p53 in H9C2 Cells

**Figure 7:**
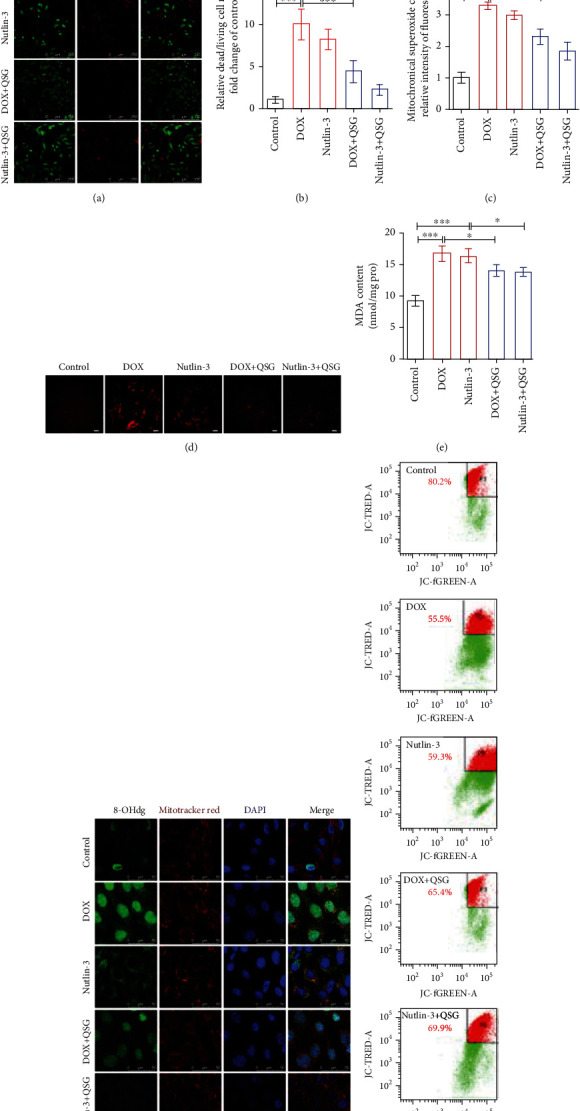
QSG reduced DOX-induced mitochondrial oxidative damage and apoptosis by suppressing p53 in H9C2 cells. (a, b) Representative images and quantitative analysis of living/dead cells by staining with calcein/PI (*n* = 6). Scale bars: 250 *μ*m. (c, d) Representative images and quantitative analysis of mitochondrial superoxide by staining with MitoSOX™ Red (*n* = 6). Scale bars: 100 *μ*m. (e) MDA content of lipid peroxidation products in cells (*n* = 4). (f) Representative images of H9C2 cells costained with 8-OHdG and MitoTracker Red. Scale bars: 50 *μ*M. (g) Quantitative analysis of mitochondrial membrane potential by JC-1 staining. ^∗^*P* < 0.05, ^∗∗∗^*P* < 0.001 vs. DOX or Nutlin-3 group. The data are shown as the mean ± s.d.

To confirm whether QSG alleviated DOX-induced mitochondrial oxidative damage and apoptosis by inhibiting p53, we applied nutlin-3 to H9C2 cells to further evaluate the changes in mitochondrial oxidative damage and apoptosis. The enhanced mean fluorescence intensity of mitosox in the DOX and nutlin-3 groups shown in Figures 7(c) and 7(d) indicated that DOX and nutlin-3 increased mitochondrial superoxide production in H9C2 cardiomyocytes, which could be reduced by QSG. The production of mitochondrial superoxide leads to lipid peroxidation of the mitochondrial membrane structure, mitochondrial DNA damage, mitochondrial membrane potential decline, and eventually, cell death. The results in Figures 7(a), 7(b), and 7(e)–7(g) show that DOX and nutlin-3 treatment of H9C2 cells resulted in a significant increase in the lipid peroxidation product MDA (Figure 7(e)), mitochondrial DNA oxidative damage product 8-OHdG (Figure 7(f)) and dead/living cell rate (Figures 7(a) and 7(b)), and a decline in the mitochondrial membrane potential (Figure 7(g)). However, QSG substantially alleviated the above mitochondrial damage and apoptosis caused by DOX and nutlin-3. Taken together, these collective data showed that activating p53 by DOX and nutlin-3 exacerbated mitochondrial superoxide production, lipid peroxidation of the mitochondrial membrane structure, mitochondrial DNA damage, mitochondrial membrane potential decline, and eventually, cell death in cardiomyocytes, which were partially rescued by QSG suppressing p53 function by increasing the binding of p53 to the MDM2 protein.

## 4. Discussion

Doxorubicin is a chemotherapeutic drug administered in adult and paediatric patients for the treatment of malignancies such as lymphoma, sarcoma, and breast cancer [33]. However, dose-dependent cardiotoxicity was recognized almost shortly after the adoption of doxorubicin treatment. When the accumulation dose of DOX is increased from 400 mg/m^2^ to 700 mg/m^2^, the incidence of heart failure will rise from 5% to 48% [34, 35]. Despite its cardiotoxicity, doxorubicin is still widely used because of the lack of suitable alternatives. Dexrazoxane, the only cardioprotectant currently approved by the US Food and Drug Administration combined with DOX [36, 37], has also been prohibited in children by the European Medicines Agency in 2011 because it induced secondary malignancies and myelosuppression [5]. This study confirmed for the first time that QSG showed an impressive cardioprotective effect in maintaining cardiac structure and function as well as mitochondrial homeostasis in DIC and provided a potential alternative for the treatment of DIC.

Excessive reactive oxygen species (ROS) generation is generally identified as the primary cause of DOX-induced cardiac dysfunction by inducing oxidative damage to mitochondria and activating the downstream proapoptotic network [7, 38, 39]. Due to the high content of mitochondria and negligible regenerative capability, myocardial mitochondria are especially vulnerable to DOX and produce abundant ROS [40], which induce mitochondrial DNA damage and lipid peroxidation of the mitochondrial membrane structure, resulting in mitochondrial dysfunction and irreversible apoptosis of cardiomyocytes [41, 42]. Similarly, our in vivo and in vitro studies also revealed that DOX induced increased myocardial ROS, mitochondrial DNA oxidative damage, and lipid peroxidation, depressed mitochondrial membrane potential, and reduced apoptosis. These findings indicate that drugs that can alleviate DOX-induced mitochondrial oxidative damage are expected to be adjuvants combined with DOX for the treatment of cancer. Encouragingly, QSG was confirmed in this study to alleviate DOX-induced damage to cardiac structure and function in mice, significantly reduce ROS and MDA production, as well as oxidative damage to mitochondrial DNA, improve mitochondrial membrane potential, and ultimately suppress apoptosis in cardiomyocytes in vivo and in vitro.

Recently, multiple mechanisms regulating mitochondrial quality control in mammals have been ascertained, including mitochondrial fission/fusion, selective mitochondrial autophagy (mitophagy) and mitochondrial biogenesis (regulating mitochondrial mass for cellular adaptation) [43, 44]. Recently, abnormalities in mitochondrial biogenesis and mitophagy have been reported in DOX-treated hearts. Among them, Parkin-mediated mitophagy, a typical and most well-studied type of mitophagy, has been reported to be suppressed due to the binding of activated p53 to the Parkin protein in the cytoplasm, resulting in blocked translocation of Parkin to mitochondria, subsequent mitochondrial dysfunction, and decreased cardiac function [19]. In addition, it has been verified that p53 activated by DOX binds to the PGC-1*α* promoter and represses the expression of PGC-1*α* [18]. PGC-1*α* is a transcriptional coactivator that modulates the gene expression of Nrf1, physically interacts with Nrf1, and coactivates the transcription of downstream TFAM, which translocates to mitochondria and activates mitochondrial DNA replication and transcription [45].

Given the above findings, our study explored the changes in mitochondrial biogenesis and mitophagy in DIC mouse hearts and the regulatory role of QSG in these changes. First, the obtained results showed that the levels of the mitochondrial biogenesis-related proteins PGC-1*α*, Nrf1, and TFAM, mitochondrial copy number, and ATP content were significantly decreased in DIC mouse myocardium. However, different doses of QSG upregulated the expression of these proteins to varying degrees and promoted mitochondrial DNA replication and ATP production. Second, we also demonstrated a significant decrease in the number of mitochondrial autophagosomes, as well as a decrease in the mitophagy protein Parkin and consequent accumulation of other mitophagy-related proteins Pink1, LC3, and p62 in the DIC mouse myocardium. Different doses of QSG improved DOX-induced mitophagy-related protein disorder and the inhibition of mitophagy.

Furthermore, we elucidated the molecular mechanisms by which DOX and QSG regulate mitophagy and mitochondrial biogenesis based on DOX-treated H9C2 cardiomyocytes. First, the obtained results showed that DOX inhibited Parkin's transfer to mitochondria by promoting the expression of p53 protein and the binding of p53 to Parkin protein, resulting in reduced distribution of Parkin, Pink1, LC3, and P62 proteins in mitochondria and accumulation in the cytoplasm. Subsequently, Parkin-mediated mitophagy was blocked, and the number of mitochondrial autophagosomes and autophagolysosomes decreased. QSG promoted the degradation of p53 by enhancing the binding of p53 to the MDM2 protein, resulting in decreased binding of p53 to the Parkin protein and increased transfer of Parkin to mitochondria. As a result, the distribution of Parkin, Pink1, LC3, and P62 proteins in mitochondria increased, which enhanced Parkin-mediated mitophagy and the formation of mitochondrial autophagosomes and autophagolysosomes. Second, we documented that DOX enhanced the inhibitory effect of p53 on PGC-1*α* protein expression as well as downstream Nrf1 and TFAM by increasing the expression of p53 protein, resulting in a significant decrease in mitochondrial DNA replication and ATP production. QSG increased the degradation of p53 by promoting the binding of p53 to the MDM2 protein, thus effectively alleviating the inhibitory effect of p53 on the expression of PGC-1*α* and downstream Nrf1 and TFAM. Finally, mitochondrial DNA replication and ATP production were significantly increased by QSG.

## 5. Conclusion

In conclusion, our study found that QSG could improve DOX-induced cardiac function decline and pathological damage to myocardia and reduce mitochondrial oxidative damage and apoptosis of myocardial cells in vivo and in vitro. More impressively, we innovatively confirmed that QSG mitigated DOX-induced mitochondrial oxidative damage and apoptosis by interfering with the protein interaction between p53 and MDM2 to modulate Parkin-mediated mitophagy and PGC-1*α*-mediated mitochondrial biogenesis.

## Figures and Tables

**Table 1:** 

Gene name	Species	Forward or 5′ primer	Reverse or 3′ primer
12S rRNA	Mouse	CCCCGCTCTACCTCACCATCTC	TCATTGGCTACACCTTGACCTAACG
	Rat	ATGCACGATAGCTAAGACCCAA	GCTGAATTAGCGAGAAGGGGTA

## Data Availability

The data used to support the findings of this study are available from the corresponding authors upon request.

## Supplementary Materials

Supplementary 1 Supplemental table 1: the antibodies used in the study.

Supplementary 2 Supplemental figure 1: the CCK-8 results of nontoxic range and effective concentration of QSG in H9C2 cells.
