# Supplementary material for: Qishen Granule Protects against Doxorubicin-Induced Cardiotoxicity by Coordinating MDM2-p53-Mediated Mitophagy and Mitochondrial Biogenesis
Source: Oxid Med Cell Longev. 2022 Sep 6;2022:4344677. doi: 10.1155/2022/4344677 (PMC9473341; doi:10.1155/2022/4344677)
Supplement: Supplementary 1 — Supplemental table 1: the antibodies used in the study. [file 4344677.f1.docx]

Table 1. The antibodies used in this paper.

| Antibodies | Companies |
| --- | --- |
| p53 | 10442-1-AP; Proteintech; United States |
| MDM2 | AF0208; Affinity Biosciences; United States |
| Parkin | 14060-1-AP; Proteintech; United States |
| Pink1 | 23274-1-AP; Proteintech; United States |
| p62 | CST88588; Cell Signaling Technology; United States |
| LC3 | 14600-1-AP; Proteintech; United States |
| PGC-1α | Bs-1832R; Bioss; United States |
| Nrf1 | CST46743; Cell Signaling Technology; United States |
| TFAM | 22586-1-AP; Proteintech; United States |
| GAPDH | CST14C10; Cell Signaling Technology; United States |
| Mouse IgG1 Isotype Control | CST5415; Cell Signaling Technology; United States |
| TOM20 | CST42406; Cell Signaling Technology; United States |
| 8-OHdG | Sc-393871; Santa Cruz Biotechnology; United States |
| VDAC1 | CST4866; Cell Signaling Technology; United States |
