# Supplementary material for: Qishen Granule Protects against Doxorubicin-Induced Cardiotoxicity by Coordinating MDM2-p53-Mediated Mitophagy and Mitochondrial Biogenesis
Source: Oxid Med Cell Longev. 2022 Sep 6;2022:4344677. doi: 10.1155/2022/4344677 (PMC9473341; doi:10.1155/2022/4344677)
Supplement: Supplementary 2 — Supplemental figure 1: the CCK-8 results of nontoxic range and effective concentration of QSG in H9C2 cells. [file 4344677.f2.docx]

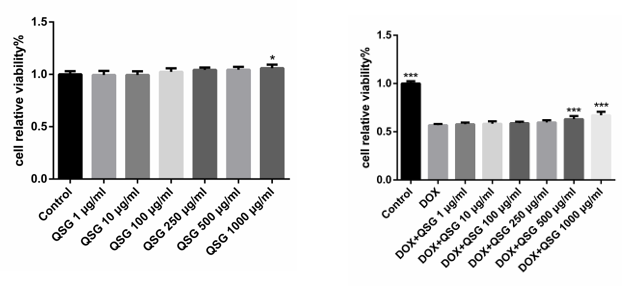


**Supplemental figure 1. The results of non-toxic range and effective concentration of QSG in H9C2 cells.**
